# A Pilot Study of the Efficacy and Economical Sustainability of Acute Coronavirus Disease 2019 Patient Management in an Outpatient Setting

**DOI:** 10.3389/fmed.2022.892962

**Published:** 2022-04-27

**Authors:** Rebecca De Lorenzo, Marco Montagna, Eleonora Bossi, Giordano Vitali, Alba Taino, Marta Cilla, Giulia Pata, Ludmilla Lazorova, Riccardo Pesenti, Chiara Pomaranzi, Cecilia Bussolari, Sabina Martinenghi, Nicoletta Bordonaro, Davide Di Napoli, Giuliano Rizzardini, Chiara Cogliati, Nuccia Morici, Patrizia Rovere-Querini

**Affiliations:** ^1^School of Medicine, Vita-Salute San Raffaele University, Milan, Italy; ^2^Unit of Hospital-Primary Care Embedding, San Raffaele Hospital, Milan, Italy; ^3^Clinical Governance Division, San Raffaele Hospital, Milan, Italy; ^4^Unit of Internal Medicine, Luigi Sacco Hospital, Azienda Socio Sanitaria Territoriale – Fatebenefratelli (ASST-FBF)-Sacco, Milan, Italy; ^5^Unit of Infectious Diseases, Luigi Sacco Hospital, Azienda Socio Sanitaria Territoriale – Fatebenefratelli (ASST-FBF)-Sacco, Milan, Italy; ^6^Istituto di Ricovero e Cura a Carattere Scientifico (IRCCS) S. Maria Nascente – Fondazione Don Carlo Gnocchi ONLUS, Milan, Italy

**Keywords:** COVID-19, outpatient clinic, healthcare, cost-effectiveness, prediction model, care pathway

## Abstract

**Objective:**

To report a preliminary experience of outpatient management of patients with Coronavirus disease 2019 (COVID-19) through an innovative approach of healthcare delivery.

**Patients and Methods:**

Patients evaluated at the Mild-to-Moderate COVID-19 Outpatient clinics (MMCOs) of San Raffaele University Hospital and Luigi Sacco University Hospital in Milan, Italy, from 1 October 2020 to 31 October 2021 were included. Patients were referred by general practitioners (GPs), Emergency Department (ED) physicians or hospital specialists (HS) in case of moderate COVID-19. A classification and regression tree (CART) model predicting ED referral by MMCO physicians was developed to aid GPs identify those deserving immediate ED admission. Cost-effectiveness analysis was also performed.

**Results:**

A total of 660 patients were included. The majority (70%) was referred by GPs, 21% by the ED and 9% by HS. Patients referred by GPs had more severe disease as assessed by peripheral oxygen saturation (SpO_2_), ratio of arterial oxygen partial pressure to fractional inspired oxygen (PaO_2_/FiO_2_), C-reactive protein (CRP) levels and interstitial involvement at lung ultrasound. Among them, 18% were addressed to the ED following MMCO assessment. CART analysis identified three independent predictors, namely home-measured SpO_2_, age and body mass index (BMI), that robustly divide patients into risk groups of COVID-19 severity. Home-measured SpO_2_ < 95% and BMI ≥ 33 Kg/m^2^ defined the *high-risk* group. The model yielded an accuracy (95% CI) of 83 (77–88)%. Outpatient management of COVID-19 patients allowed the national healthcare system to spare 1,490,422.05 € when compared with inpatient care.

**Conclusion:**

Mild-to-moderate COVID-19 outpatient clinics were effective and sustainable in managing COVID-19 patients and allowed to alleviate pressure on EDs and hospital wards, favoring effort redirection toward non-COVID-19 patients.

## Introduction

Coronavirus disease 2019 (COVID-19) pandemic has posed significant challenges on healthcare systems worldwide due to an overwhelming surge of patients simultaneously seeking medical care (1, 2). Emergency departments (ED) probably suffered the most, being the bottleneck of patients with acute disease, often regardless of symptom severity. In fact, a proportion of patients presenting to the ED had mild to moderate clinical features not requiring urgent care or hospital admission (3, 4).

While patients with mild disease and no risk factors for progression may benefit from medical assistance by general practitioners (GPs), those with moderate COVID-19 or harboring risk factors for adverse outcomes reside in a gray area between in-hospital and home management (5). Within the latter patient category, GPs may not have the tools to discriminate nor handle patients deserving more attentive monitoring. On the other hand, unfiltered hospital referral of these patients may cause unjustified ED overcrowding and saturation of hospital beds. Accurate patient evaluation in a hospital-based outpatient setting by expert physicians may fill this gap, allowing for timely risk classification and informed management decision-making.

On the heels of the first pandemic wave and with the belief that some measure had to be taken to avoid system collapse, health policymakers of Lombardy region in Italy at the beginning of the second wave designed an integrated approach of healthcare delivery, called “Hot Spot” or “Mild-to-moderate COVID-19 outpatient clinic” (MMCO), based on the strict, bidirectional collaboration with GPs and the ED (5). One year after the introduction of this novel service, here we describe our preliminary experience of patient management at two MMCOs of the metropolitan city of Milan, specifically those of San Raffaele University Hospital and Luigi Sacco University Hospital. Moreover, we provide an evidence-based tool for patient classification into risk groups by the GP beforehand, to identify patients deserving early ED referral with the aims of optimizing patient management and spare resources.

## Materials and Methods

### Mild-to-Moderate COVID-19 Outpatient Clinic Organization and Patient Referral

**FIGURE 1:**
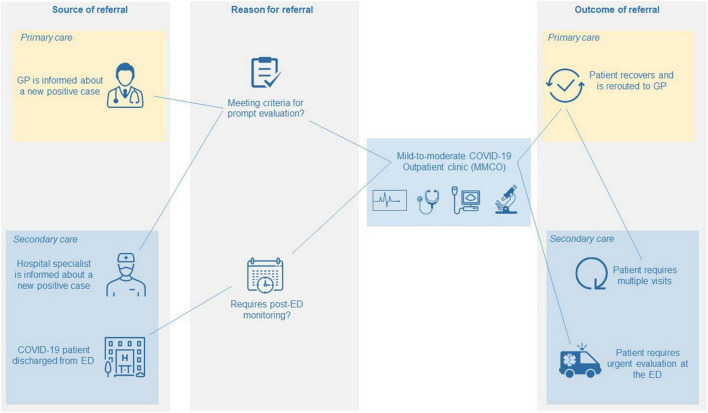
Care pathway of patients at MMCOs from referral to discharge. GP, general practitioner; ED, emergency department.

Mild-to-moderate COVID-19 outpatient clinic organization and the process of patient flow from referral to discharge is described in Figure 1. MMCOs are located within hospitals, in a strategic location that is both easy-to-reach by patients and in direct connection with the ED. This innovative healthcare service is addressed to two different categories of patients with nasopharyngeal swab-confirmed infection: (i) patients with moderate COVID-19 and (ii) patients at increased risk of adverse outcome due to pre-existing risk factors independent of COVID-19 severity. Both categories may need active surveillance and management by physicians with an established expertise in treating COVID-19 and its complications. Patients can be referred to the MMCO by GPs, ED physicians or hospital specialists (HS) through direct telephone call to the MMCO physician at a dedicated mobile number, which is active 12 h per day, 7 days per week.

Mild-to-moderate COVID-19 outpatient clinic physicians are internal medicine doctors. Criteria for referral to MMCOs of patients with moderate COVID-19 by GPs are derived from official regional regulations (5, 6). Prior to patient evaluation at the MMCO, the GP provides the MMCO physician with a comprehensive report, in the form of a standardized questionnaire (Supplementary Figure 1), on the patient’s past medical history, COVID-19-related symptoms, time from symptom onset, peripheral oxygen saturation (SpO_2_), body mass index (BMI), chronic therapies and COVID-19-specific treatments. Criteria for referral of include: (i) age ≥65 years in the presence of body temperature ≥38°C and at least two comorbidities among obesity, active cancer, chronic kidney disease (CKD), chronic respiratory disease, immunosuppression, ischemic heart disease (IHD), diabetes mellitus (DM), coagulopathy, history of immunosuppression or organ transplant, HIV infection and cerebrovascular disease (CVD); (ii) body temperature ≥38°C for longer than 72 h; (iii) SpO_2_ between 90 and 94% (or between 88 and 90% in case of history of chronic obstructive pulmonary disease).

Emergency department physicians may refer patients who may benefit from prolonged monitoring in a hospital-based setting following clinical stabilization, with the dual purpose of relieving the ED from excessive burden and limiting hospitalization rates, while feeling at ease discharging patients with a non-negligible risk of disease evolution.

Hospital specialists, usually hematologists or oncologists, may especially benefit from extending referral to asymptomatic or mild COVID-19 patients in case of pre-existing risk factors for poor clinical outcome (i.e., cancer or other frailty conditions).

Following the first evaluation, patients may either be discharged from the MMCO and redirected to GP care, or be addressed to the ED in case of severe COVID-19 requiring more intense care, or be scheduled for further visits for a prolonged monitoring at the MMCO. Specifically, active surveillance at the MMCO consists of serial visits, at varying time intervals depending on disease severity, until disease stabilization or complete recovery.

### Patient Evaluation at Mild-to-Moderate COVID-19 Outpatient Clinics

The first visit at MMCO comprises a comprehensive physical examination with vital sign assessment (SpO_2_, heart and respiratory rates, blood pressure, body temperature, blood glucose) and measurement of anthropometric parameters including weight, height and waist circumference. Data about past and COVID-19-related medical history are accurately collected, integrating patient interview with the GP’s questionnaire and available medical records. Lung assessment relies on lung ultrasound (LUS) imaging. In addition to being easy and rapid to perform, LUS has higher sensitivity and specificity for lung parenchymal abnormalities than chest X-rays (7–9). Moreover, it can be performed at bedside and bears no radiological hazard (8). Through LUS, signs of interstitial lung disease including white lung pattern suggestive of more severe involvement and parenchymal consolidations may be detected. Also, LUS allows to calculate the Lung UltraSound Score (LUSS), a semi-quantitative score of lung aeration loss (10, 11), which has been associated with disease severity and mortality in COVID-19 (12, 13). Arterial blood gas analysis parallels LUS in the evaluation of pulmonary dysfunction, and the ratio of arterial oxygen partial pressure (PaO_2_, in mmHg) to fractional inspired oxygen (FiO_2_, in mmHg), expressed as a fraction, is used as a quantitative marker of respiratory insufficiency. Electrocardiography at rest and blood exams including complete blood count, C reactive protein (CRP), lactate dehydrogenase (LDH), D-dimer, ferritin and creatinine are also performed.

At the following visits, the abovementioned procedures may be repeated in varying combinations to allow for an individualized and attentive disease monitoring.

### Study Design

All patients aged 18 years or older, evaluated at the MMCOs of San Raffaele University Hospital and Luigi Sacco University Hospital in Milan, Italy, from 1 October 2020 to 31 October 2021 were included in the present study.

Data were retrospectively collected as part of the retroPAUCI protocol (N. 140/INT/2021), approved by the Hospital Ethics Committees, in conformity to the declaration of Helsinki. Written informed consent was obtained by all patients.

### Variables

Age, sex, past medical history (i.e., obesity, active cancer, CKD, chronic respiratory disease, immunosuppression, IHD, DM, coagulopathy, history of immunosuppression or organ transplant, HIV infection, CVD), BMI, COVID-19-related history including time of symptom onset, COVID-19-related symptoms (i.e., dyspnea, cough, taste and smell disturbances, pharyngodynia, myalgias, arthralgias, asthenia, diarrhea, nausea or vomiting, headache, syncope), home-measured SpO_2_ at time of MMCO referral and presence of fever for ≥72 h were collected for all patients. Recorded data on patient evaluation during the first MMCO visit comprised blood pressure, heart rate, SpO_2_, respiratory rate (RR), PaO_2_/FiO_2_ at arterial blood gas analysis, blood exams (i.e., absolute neutrophil and lymphocyte counts, neutrophil to lymphocyte ratio [NLR], LDH, CRP, creatinine, ferritin and D-dimer), as well as LUSS, the presence of white lung pattern or parenchymal consolidations at LUS. Moreover, rates of ED referral following MMCO evaluation and of hospitalization after ED admission, observation time (i.e., time interval from the first MMCO visit to MMCO discharge), and the number of visits at MMCO prior to discharge were also registered.

Prior to analysis, data were cross-checked with medical charts and verified by data managers and clinicians for accuracy.

### Primary Outcome

To investigate which patients are at increased risk of adverse outcome, ED referral following MMCO evaluation was used as primary outcome.

### Cost-Effectiveness Analysis

We investigated whether managing patients at MMCO was economically convenient for the hospital compared with inpatient care. We considered that patients who received ≥2 MMCO visits would have otherwise been hospitalized due to the need of active surveillance. Therefore, for the purpose of the analysis, the number of patients who performed ≥2 MMCO visits was used to define the number of spared hospitalizations. The cost of one hospital stay for COVID-19 was computed as the weighted mean of the hospitalization costs for all COVID-19 patients hospitalized during the same time interval (i.e., October 2020–October 2021), not transferred to the intensive care unit. Specifically, the cost of each hospitalization was estimated based on the International Classification of Diseases, Ninth Revision, Clinical Modification (ICD-9-CM) codes for diagnoses and procedures linked to COVID-19, according to the updated guidelines of Regional Health Authorities (14, 15). On the other hand, the overall cost for 1-year activity at the MMCO was calculated taking into account: (i) cost of the personnel (two medical doctors, one nurse, one clerk), (ii) cost of consumables (personal protective equipment, sanitary ware, stationery), (iii) cost of general utilities, building and instrument (i.e., electrocardiography, ultrasound and arterial blood gas analysis machinery) maintenance, etc., (iv) indirect hospital-related costs. Supplementary Table 1 describes in detail how the total cost of activity at MMCO during the study time was calculated.

### Statistical Analyses

Descriptive statistics were performed for all variables. Dichotomous variables were expressed as absolute counts (percentage), and continuous variables as medians (interquartile range, IQR) unless differently specified. χ^2^ test and Kruskal–Wallis test were used to perform group comparisons for categorical and continuous variables, respectively.

To identify early predictors of adverse outcome (i.e., ED referral following MMCO evaluation) and provide GPs with a tool for early risk classification, we employed a classification and regression tree (CART) algorithm within the cohort of patients referred to the MMCO by GPs. CART analysis relies on recursive partitioning to sequentially split a cluster of patients into homogeneous sub-groups based on independent variables, determining the hierarchy of prognostic factors and associated cut-points that best subdivides the initial population to obtain faithful risk groups (16, 17). Demographical data, comorbidities, BMI, and parameters that GPs can easily obtain through patient interview, including home-measured SpO_2_, the presence of fever for ≥72 h, COVID-19-related symptoms and time from symptom onset were included as predictors in the CART. The results of the analysis were graphically represented. The area under the receiver operating characteristic (ROC) curve (AUC) was used as a quality metric of the CART.

Missing data was not imputed.

All statistical analyses were performed using R statistical package (version 4.0.0, R Foundation for Statistical Computing, Vienna, Austria), with a two-sided significance level set at *p* < 0.05.

## Results

### Patient Characteristics and Sources of Mild-to-Moderate COVID-19 Outpatient Clinic Referral

From 1 October 2020 to 31 October 2021, a total of 660 patients were evaluated at the MMCOs of San Raffaele University Hospital and Luigi Sacco University Hospital. The total number of visits was 1101 and their distribution within the study time interval is depicted in Supplementary Figure 2. Baseline patient characteristics and indicators of COVID-19 severity at MMCO evaluation are reported in Tables 1, 2, respectively. The source of MMCO referral was known for 572 patients. Of these, 400 (70%) were referred by GPs, 119 (21%) by the ED and 53 (9%) by HS.

**TABLE 1:** General characteristics of COVID-19 patients evaluated at the mild-to-moderate COVID-19 outpatient clinic.

Variable	Overall (*n* = 660)	Source of MMCO referral			*P*-value
		
		GP (*n* = 400)	ED (*n* = 119)	Hospital specialist (*n* = 53)	
Age (years)	56 (46–66)	56 (49–68.2)	53 (42–66.5)	55 (48–63)	0.080
Female sex	284 (43)	171 (42.8)	45 (37.8)	25 (47.2)	0.47
BMI (Kg/m^2^)	26 (23–29)	25.4 (23.1–28.4)	27.1 (23–30.2)	26 (23.3–28)	0.20
**Comorbidities**					
Obesity (BMI ≥ 30)	119 (18)	66 (16.5)	33 (27.7)	7 (13.2)	0.029
DM	59 (8.9)	37 (9.2)	11 (9.2)	3 (5.7)	0.68
Active cancer	79 (12)	35 (8.8)	14 (11.8)	16 (30.2)	<0.0001
CKD	13 (2)	6 (1.5)	1 (0.8)	3 (5.7)	0.066
CRD	49 (7.4)	38 (9.5)	9 (7.6)	1 (1.9)	0.16
IHD	34 (5.2)	21 (5.2)	5 (4.2)	5 (9.4)	0.36
CVD	12 (1.8)	10 (2.5)	0 (0)	2 (3.8)	0.17
HIV infection	4 (0.6)	2 (0.5)	1 (0.8)	0 (0)	0.77
Coagulopathies	14 (2.1)	12 (3)	2 (1.7)	0 (0)	0.42
Immunosuppression or organ transplant	18 (2.7)	4 (1)	1 (0.8)	7 (13.2)	<0.0001
**COVID-19-related history**					
Time from symptom onset to MMCO referral (days)	10 (7–13)	9 (7–12)	12 (9–16)	10 (6–16)	<0.0001
Home-measured	96.2 (0.107)	95.7 (0.128)	96.1 (0.256)	97.2 (0.327)	<0.0001
SpO_2_ (%)	96 (94–98)	96 (94–98)	96 (94–98)	97 (95.5–99.5)	0.00011
Fever for ≥ 72 h	221 (33.5)	173 (43.2)	32 (26.9)	12 (22.6)	0.0026
Dyspnea	425 (64.4)	243 (60.8)	78 (65.5)	28 (52.8)	0.18
Cough	510 (77.3)	319 (79.8)	97 (81.5)	40 (75.5)	0.29
Taste disturbance	168 (25.5)	90 (22.5)	29 (24.4)	17 (32.1)	0.54
Smell disturbance	163 (24.7)	86 (21.5)	28 (23.5)	16 (30.2)	0.58
Pharyngodynia	121 (18.3)	66 (16.5)	23 (19.3)	9 (17)	0.62
Myalgias	313 (47.4)	187 (46.8)	55 (46.2)	28 (52.8)	0.92
Arthralgias	269 (40.8)	166 (41.5)	44 (37)	26 (49.1)	0.81
Asthenia	435 (65.9)	274 (68.5)	75 (63)	37 (69.8)	0.35
Diarrhea	187 (28.3)	102 (25.5)	35 (29.4)	15 (28.3)	0.47
Nausea/vomiting	136 (20.6)	77 (19.2)	30 (25.2)	7 (13.2)	0.055
Headache	229 (34.7)	130 (32.5)	33 (27.7)	21 (39.6)	0.84
Syncope	11 (1.7)	7 (1.8)	3 (2.5)	0	0.47

*MMCO, mild-to-moderate COVID-19 outpatient clinic; GP, general practitioner; ED, emergency department; BMI, body mass index; SpO_2_, peripheral oxygen saturation; DM, diabetes mellitus; CKD, chronic kidney disease; CRD, chronic respiratory disease; IHD, ischemic heart disease; CVD, cerebrovascular disease; HIV, Human immunodeficiency virus.*

*Dichotomous variables were expressed as count (percentage) and continuous variables as median (interquartile range). Home-measured SpO_2_ (%) was expressed as both mean (± standard deviation) and median (interquartile range).*

**TABLE 2:** Health status and indicators of disease severity in COVID-19 patients evaluated at the mild-to-moderate COVID-19 outpatient clinic.

Variable	Overall (*n* = 660)	Source of MMCO referral			*P*-value
		
		GP (*n* = 400)	ED (*n* = 119)	Hospital specialist (*n* = 53)	
Systolic BP (mmHg)	133 (121–145)	133 (1201–145)	130 (120–141)	138 (120–143)	0.71
Diastolic BP (mmHg)	80 (75–87)	80 (75–88)	80 (75–86)	80 (73–89)	0.97
Heart rate (bpm)	85 (75–95)	85 (75–94)	83 (73–92)	86 (75–97)	0.22
SpO_2_ (%)	97.2 (0.114)	96.9 (0.15)	97.4 (0.223)	98.2 (0.257)	0.00072
	98 (96–99)	98 (96–99)	98 (97–99)	98 (98–100)	0.00053
RR (breaths/min)	20 (16–22)	20 (17–22)	20 (18–24)	18 (16–20)	0.073
PaO_2_/FiO_2_	362 (327–403)	356 (325–395)	370 (326–407)	398 (346–426)	0.027
**Blood exams**					
CRP (mg/dL)	14 (4–37)	18 (6–53)	13 (5–24)	3 (1–14)	0.0028
LDH (U/L)	245 (207–304)	251 (215–312)	238 (203–311)	237 (216–260)	0.35
D-dimer (ng/L)	0.5 (0.4–0.8)	0.5 (0.4–0.8)	0.5 (0.3–0.7)	0.5 (0.3–1.1)	0.73
Neutrophils (x10^9^/L)	4 (2.6–5.9)	3.9 (2.7–5.5)	4.6 (3.5–6.7),	3.5 (2.4–4.7)	0.0087
Lymphocytes (x10^9^/L)	1.3 (0.9–1.8)	1.2 (0.9–1.6)	1.3 (0.8–2)	2 (1.3–2.5)	0.0089
NLR	3 (1.8–5.7)	3.1 (1.8–4.6)	3.4 (1.8–7.5)	1.7 (1–2.7)	0.0013
Ferritin (ng/mL)	391 (161–777)	392 (182–748)	429 (183–696)	373 (157–1180)	0.95
Creatinine (mg/dL)	0.9 (0.8–1.1)	0.9 (0.8–1.1)	0.9 (0.8–1)	0.8 (0.7–1.1)	0.51
**Lung ultrasound**					
LUSS	5 (2–9)	5.5 (2–10)	5 (3–9)	3 (0–7)	0.035
White lung pattern	303 (45.9)	203 (50.7)	49 (41.2)	19 (35.8)	0.0098
Parenchymal consolidation	123 (18.6)	61 (15.2)	28 (23.5)	4 (7.5)	0.026
ED referral	97 (14.7)	73 (18.2)	7 (5.9)	6 (11.3)	0.0030
ED referral at 1st MMCO visit	77 (11.7)	61 (15.2)	6 (5)	5 (9.4)	0.0053
ED referral at 2nd MMCO visit	20 (3)	11 (2.8)	1 (0.8)	1 (1.9)	0.55
≥2 MMCO visits^#^	235 (41.7)	131 (40.1)	54 (48.2)	18 (38.3)	0.28
Observation time (days)^#^, ^§^	4.8 (0.40)	4.8 (0.57)	4.9 (0.61)	5.5 (1.34)	0.66
	1 (0–7)	0 (0–7)	4 (0–8)	0 (0–8)	0.38

*MMCO, mild-to-moderate COVID-19 outpatient clinic; GP, general practitioner; ED, emergency department; BP, blood pressure; bpm, beats per minute; SpO_2_, peripheral oxygen saturation; RR, respiratory rate; PaO_2_/FiO_2_, arterial oxygen partial pressure/fractional inspired oxygen; CRP, C reactive protein; LDH, lactate dehydrogenase; NLR, neutrophil to lymphocyte ratio; LUSS, lung ultrasound score.*

*Source of MMCO referral was known for 572 patients.*

*Dichotomous variables were expressed as count (percentage) and continuous variables as median (interquartile range). SpO_2_ (%) and observation time (days) were expressed as both mean (± standard deviation) and median (interquartile range).*

*^#^Excluding patients referred to the ED by MMCO physicians.*

*^§^Calculated as the time from the first MMCO visit to discharge from the MMCO. An observation time of 0 indicates that a patient was discharged following the first MMCO visit.*

Most patients were male and median age was 56 (46–66) years. Median BMI was in the overweight range, 18% of patients being obese. Obesity was more common in patients discharged from the ED (27.7 vs. 16.5% in patients referred by GPs and 13.2% in those referred by HS, p 0.029). Except for active cancer and immunosuppression or history of organ transplant, which were, as expected, significantly more common in patients referred by HS (both *p* < 0.0001), no difference among the three groups was recorded in terms of other comorbidities (Table 1).

With regard to COVID-19-related history, time from symptom onset to MMCO was shorter in patients referred by GPs (9 [7–12] vs. 12 [9–16] in patients discharged from the ED and 10 [6–16] in those referred by HS, *p* < 0.0001). No difference was observed in terms of COVID-19-related symptoms, the most common complaint being cough in all groups. Home-measured SpO_2_ was significantly lower in these patients (mean [standard deviation, SD] 95.7 [0.128]) compared to patients referred by the ED (96.1 [0.256]) or by HS (97.2 [0.327], *p* < 0.0001). Patients referred by GPs also more frequently reported fever for ≥72 h (43.2 vs. 26.9% in patients referred by the ED and 22.6% in those referred by HS, p 0.0026, Table 1).

Overall, patients referred by GPs had more severe COVID-19 clinical features at MMCO evaluation than the other two groups. Specifically, both SpO_2_ and PaO_2_/FiO_2_ were significantly reduced in these patients (both *p* < 0.05), while those referred by HS registered the highest values in line with their expected milder clinical features. Similarly, CRP levels were significantly increased in patients referred by GPs (18 [6–53] vs. 13 [5–24] in patients discharged from the ED and 3 [1–14] in those referred by HS, p 0.0028). At LUS, more patients in the group referred by GPs had white lung pattern (50.7 vs. 41.2% in patients discharged from the ED and 35.8% in those referred by HS, p0.0098) and median LUSS was higher in this group (5.5 [2–10], p 0.035). Parenchymal consolidation was instead a more common finding in patients discharged from the ED (23.5 vs. 15.2% in patients referred by GPs and 7.5% in those referred by HS, p 0.026, Table 2).

### Clinical Outcome Following Mild-to-Moderate COVID-19 Outpatient Clinic Evaluation

Following patient assessment at the MMCO, 97 out of 660 patients (15%) were referred to the ED for an urgent shift toward more intense care. Specifically, 77 (79%) patients were addressed to the ED soon after the first MMCO visit, while 20 (21%) following the second visit. Of the 400 patients referred to MMCO by GPs, 73 (18%) were addressed to the ED, compared to 11% (6 of 53) of those referred by HS. A minority of patients discharged by the ED (7 of 119 [6%]) were redirected to the ED following MMCO evaluation. Rates of hospitalization following ED admission were 66% (48 out of 73), 71% (5 out of 7) and 67% (4 out of 6) in patients initially referred to the MMCO by GPs, ED, and HS, respectively.

Excluding patients addressed to the ED following MMCO visit, 235 out of 563 patients (42%) were scheduled for at least one additional MMCO visit due to the need of continued hospital-based monitoring, while 328 (58%) were discharged after the first evaluation and redirected to GP care due to mild COVID-19.

### Risk Classification Algorithm for the Need of Early Emergency Department Referral

In light of the observation that patients addressed to the MMCO by GPs had overall more severe COVID-19 at MMCO evaluation, we hypothesized that some of these patients might benefit from early ED referral directly by the GP, prior to MMCO visit. Therefore, we aimed at providing GPs with an evidence-based tool able to identify high-risk patients prior to MMCO evaluation, avoiding unnecessary time lags.

**FIGURE 2:**
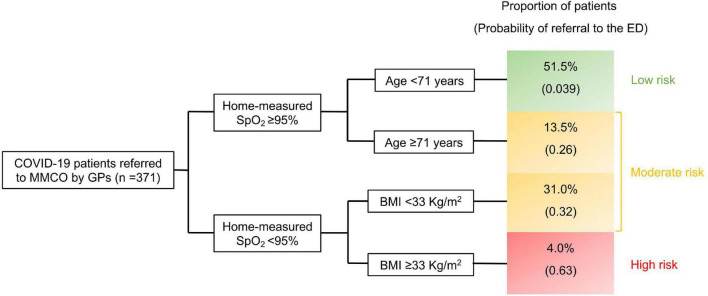
Classification and regression tree (CART) model predicting emergency department referral by the physician of the mild-to-moderate COVID-19 outpatient clinic (MMCO). MMCO, mild-to-moderate COVID-19 outpatient clinic; GP, general practitioner; SpO_2_, peripheral oxygen saturation; BMI, body mass index; ED, emergency department.

**FIGURE 3:**
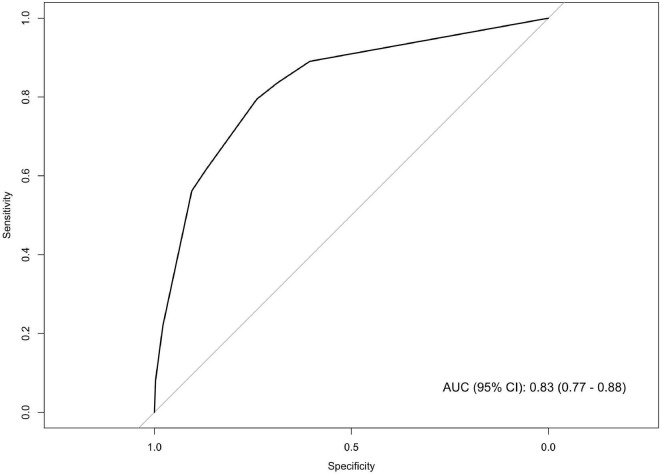
Receiver operating curve (ROC) of the classification and regression tree (CART) model predicting emergency department referral by the physician of the mild-to-moderate COVID-19 outpatient clinic (MMCO). AUC, area under the ROC curve.

As mentioned above, among the totality of patients referred by GPs (*n* = 400), 18% were addressed to the ED by the MMCO physician due to the need of more intense hospital-based assistance. We used CART analysis to build an easy-to-use algorithm that exploits parameters obtainable by simple patient interview. Among demographics, comorbidities, BMI, home-measured SpO_2_, the presence of fever for ≥72 h, COVID-19-related symptoms and time from symptom onset, CART analysis selected three variables, namely home-measured SpO_2_, age and BMI, able to robustly classify patients into risk groups for the early need of intense care. Moreover, for each of these variables, it identified the thresholds that maximized the segregation among the resulting patient clusters (Figure 2). Three risk groups were obtained: (i) *low risk* (home-measured SpO_2_ ≥ 95% and age < 71 years), (ii) *moderate risk* (home-measured SpO_2_ ≥ 95% and age ≥ 71 years or home-measured SpO_2_ < 95% and BMI < 33 Kg/m^2^), and (iii) *high risk* (home-measured SpO_2_ < 95% and BMI ≥ 33 Kg/m^2^). The AUC (95% confidence interval, CI) of the ROC for the CART (Figure 3) was 0.83 (0.77–0.88).

The accuracy of the CART model was subsequently confirmed when comparing the identified risk groups in terms of indicators of disease severity assessed during MMCO evaluation. In fact, patients in the *high-risk* group had overall more severe COVID-19 than those in the *moderate*- and *low-risk* groups, differences being expectedly more pronounced compared with the *low-risk* group (Table 3). Specifically, SpO_2_ (%, 93 [91–94] in *the high-risk* group vs. 97 [95–98] in the *moderate-risk* and 98 [97–99] in the *low-risk* groups) and PaO_2_/FiO_2_ (314 [289–341] in *the high-risk* group vs. 348 [303–373] in the *moderate-risk* and 371 [339–409] in the *low-risk* groups) were significantly reduced in *high-risk* patients (both *p* < 0.0001), in parallel to a significant increase in RR (breaths/min, 22 [20–24] in *the high-risk* group vs. 20 [18–22] in the *moderate-risk* and 18 [16–21] in the *low-risk* groups, *p* < 0.0001). Similarly, blood levels of CRP and LDH, as well as NLR were significantly higher in patients in the *high-risk* group (all *p* < 0.05). Likewise, absolute lymphocyte count was significantly reduced in *high-risk* patients (p 0.0023), in line with a more severe disease. Ferritin showed a tendency toward being increased in the *high-risk* group (p 0.052). At LUS, white lung pattern was more common in patients in the *moderate*- and *high-risk* groups compared to the *low-risk* cluster, while no difference was observed in the rate of parenchymal consolidation. LUSS was significantly lower in the *low-risk* group compared to the other groups.

**TABLE 3:** Indicators of disease severity in COVID-19 patients referred to the mild-to-moderate COVID-19 outpatient clinic by GPs according to the risk of early ED referral.

Variable	Overall (*n* = 400)	Low risk (*n* = 186)	Moderate risk (*n* = 167)	High risk (*n* = 18)	*P*-value
SpO_2_ (%)	96.9 (0.15)	98 (0.183)	96.2 (0.214)	93.2 (0.639)	<0.0001
	98 (96–99)	98 (97–99)	97 (95–98)	93 (91–94)	<0.0001
RR (breaths/min)	20 (17–22)	18 (16–21)	20 (18–22)	22 (20–24)	<0.0001
PaO_2_/FiO_2_	356 (325–395)	371 (339–409)	348 (303–373)	314 (289–341)	<0.0001
**Blood exams**					
CRP (mg/dL)	18 (6–53)	12 (2–28)	35 (14–71)	38 (29–56)	0.00017
LDH (U/L)	251 (215–312)	249 (209–294)	247 (216–312)	376 (329–394)	0.029
D-dimer (ng/L)	0.5 (0.4–0.8)	0.5 (0.4–0.7)	0.6 (0.4–0.9)	0.7 (0.4–0.8)	0.26
Neutrophils (x10^9^/L)	3.9 (2.7–5.5)	3.3 (2.4–4.6)	4.4 (3–6)	5.5 (3.9–5.6)	0.030
Lymphocytes (x10^9^/L)	1.2 (0.9–1.6)	1.3 (1.1–1.8)	1.1 (0.9–1.3)	0.7 (0.7–0.8)	0.0023
NLR	3.1 (1.8–4.6)	2.4 (1.6–3.7)	3.5 (2.3–6.5)	6.9 (3.1–8)	0.0013
Ferritin (ng/mL)	392 (182–748)	351 (138–647)	428 (249–819)	614 (539–1427)	0.052
Creatinine (mg/dL)	0.9 (0.8–1.1)	0.9 (0.8–1)	1 (0.8–1.2)	1 (0.9–1)	0.019
**Lung ultrasound**					
LUS	6 (2–10)	4 (1–8)	7 (4–11)	6 (4–9)	<0.0001
White lung pattern	203 (50.7)	84 (45.2)	106 (63.5)	10 (55.6)	0.0043
Parenchymal consolidation	61 (15.2)	26 (14)	31 (18.6)	3 (16.7)	0.54

*MMCO, mild-to-moderate COVID-19 outpatient clinic; GP, general practitioner; ED, emergency department; SpO_2_, peripheral oxygen saturation; RR, respiratory rate; PaO_2_/FiO_2_, arterial oxygen partial pressure/fractional inspired oxygen; CRP, C reactive protein; LDH, lactate dehydrogenase; NLR, neutrophil to lymphocyte ratio; LUS, lung ultrasound score.*

*The risk was calculated for 371 patients based on data availability.*

*Dichotomous variables were expressed as count (percentage) and continuous variables as median (interquartile range). SpO_2_ (%) was expressed as both mean (± standard deviation) and median (interquartile range).*

### Cost-Effectiveness of Mild-to-Moderate COVID-19 Outpatient Clinic

A total of 235 out of 660 (41.7%) patients performed ≥2 visits at MMCO and were thus included in the cost-effectiveness analysis. Based on the ICD-9-CM and the updated guidelines of Regional Health Authorities codes for diagnoses and procedures linked to COVID-19, the cost of one COVID-19-related hospitalization was estimated to be 3,275.86 €. According to the regional reform of 2021 on the increased pricing for the activities provided to COVID-19 patients, the cost of each hospitalization was increased by 3,713 € (14, 15). Therefore, the total mean cost of one hospitalization for COVID-19 was estimated as being 6,988.86 € (3,275.86 + 3,713 €). Considering that patients receiving ≥2 MMCO evaluations would have most likely been hospitalized to continue hospital-based active surveillance, we calculated the theoretical total cost of hospitalizations by multiplying 6,988.86 € times 235 patients, resulting in 1,642,382.10 €. On the other hand, considing the totality of visits performed at MMCOs (*n* = 1101), the overall cost of MMCO activity during the study period was estimated as being 151,960.05 € (Supplementary Table 1). Therefore, the total amount of euros spared through the management of COVID-19 patients at MMCOs rather than by hospital admission during the period October 2020-October 2021 was 1,490,422.05 (i.e., 1,642,382.10–151,960.05 €).

## Discussion

Here we describe an innovative healthcare strategy to optimize the management system of COVID-19 patients while sparing resources for the care of patients with non-COVID-19-related conditions. Similar models have previously been proposed (18, 19). MMCOs were designed to fill the gap of care delivery to COVID-19 patients with clinical features that are neither too mild to be managed by the GP in a home-based setting nor too severe to require ED admission or hospitalization. In our experience, these novel infrastructures allowed the achievement of the dual goal of chaperoning GPs in the management of COVID-19 patients and alleviating pressure on EDs and hospital wards, favoring effort redirection toward patients affected by other conditions. Indeed, the first wave of COVID-19 pandemic forced an extensive reduction of several non-COVID-19-related activities to the detriment of non-COVID-19 care (20–22). The success of this approach dwells in the high degree of inter-system coordination and commitment to the integration of hospital and primary care services.

In a timespan of 1 year, two MMCOs in Milan took care of hundreds of patients who would otherwise be directed straightforwardly to the ED due to the intrinsic difficulty of GPs to deliver optimal care in the absence of hospital equipment or, perhaps, COVID-19 expertise. Most of these patients were indeed managed at MMCOs for the entire course of their disease through serial visits, always in strict collaboration with GPs, until clinical recovery. Only a minority of patients, specifically less than 15%, were addressed to the ED for an urgent evaluation in an emergency setting due to severe disease or high risk of disease progression. Noteworthily, the majority of these patients (65%) required hospitalization following ED admission, pointing to the high level of appropriateness of clinical decisions by MMCO physicians.

Considering that GPs may dispose of insufficient instruments to discriminate patients at increased risk of adverse outcome (23), in light of our observation that a higher proportion of patients among those referred by GPs than those referred by ED or HS were addressed to the ED following MMCO visit, we speculated that the early identification by GPs of patients deserving direct ED admission might guarantee proper and timely intervention. Therefore, we developed an evidence-based, easy-to-use tool that GPs can employ during patient interview to identify patients at high risk of disease progression. CART analysis, through a machine-learning approach, selected three variables, namely home-measured SpO_2_, age and BMI as the independent predictors that most robustly divide patients into faithful risk groups for severe disease. The model yielded an AUC of 83%, far above the ideal accuracy threshold of 70%. The predictive strength of the model was confirmed by subsequent analysis showing that patients in the *high-risk* group were indeed those who exhibited the highest degree of respiratory insufficiency, as measured by SpO_2_, RR and PaO_2_/FiO_2_, and the worse laboratory findings. Also, LUS demonstrated a decreased rate of interstitial abnormalities in patients in the *low-risk* group.

In addition to the clinical efficacy of MMCO in terms of support to GPs and relief on ED and hospital wards, the cost-effectiveness analysis showed that the proposed model of COVID-19 outpatient management is also economically sustainable for the National Healthcare System. Caution is, however, warranted in interpreting economical results, given that many factors besides COVID-19 diagnosis may influence the decision of hospital admission and the length of hospital stay. Nonetheless, outpatient management of COVID-19 patients should be preferred when feasible.

Overall, the establishment of MMCOs proved to be a deal-breaker for the management of COVID-19 patients in a sustainable and efficient way. Ideally, MMCOs may also serve as safe environments where candidate patients might receive COVID-19-directed therapies such as anti-SARS-CoV-2 monoclonal antibodies, antiviral therapies, etc., under the expert monitoring of trained personnel. This patient-centered, sustainable and flexible approach would ensure continuity of care through a 360-degree assistance and possibly serve as a template beyond COVID-19 outbreak.

## Data Availability Statement

The raw data supporting the conclusions of this article will be made available by the authors, without undue reservation.

## Ethics Statement

The studies involving human participants were reviewed and approved by the Comitato Etico Ospedale San Raffaele. The patients/participants provided their written informed consent to participate in this study.

## Author Contributions

RD and MM: conception and design, acquisition of data, analysis and interpretation of data, statistical analyses, and drafting of the manuscript. EB, GV, AT, MC, GP, LL, RP, CP, CB, SM, NB, DD, and GR: acquisition of data and critical revision of the manuscript. CC, NM, and PR-Q: conception and design, interpretation of data, drafting of the manuscript, and supervision. All authors contributed to manuscript revision, read, and approved the submitted version.

## Conflict of Interest

The authors declare that the research was conducted in the absence of any commercial or financial relationships that could be construed as a potential conflict of interest.

## Publisher’s Note

All claims expressed in this article are solely those of the authors and do not necessarily represent those of their affiliated organizations, or those of the publisher, the editors and the reviewers. Any product that may be evaluated in this article, or claim that may be made by its manufacturer, is not guaranteed or endorsed by the publisher.

## Supplementary Material

Supplementary Figure 1 Form of patient referral to the Mild-to-Moderate COVID-19 Outpatient Clinic by General Practitioners. SpO_2_, peripheral oxygen saturation; BMI, body mass index; HIV, Human Immunodeficiency Virus.

Supplementary Figure 2 Distribution over time of COVID-19 patient visits at Mild-to-Moderate COVID-19 Outpatient Clinics.

## References

[1] Armocida B Formenti B Ussai S Palestra F Missoni E. The Italian health system and the COVID-19 challenge. *Lancet Public Health.* (2020) 5:e253. 10.1016/S2468-2667(20)30074-8 PMC7104094 32220653

[2] Miller IF Becker AD Grenfell BT Metcalf CJE. Disease and healthcare burden of COVID-19 in the United States. *Nat Med.* (2020) 26:1212–7. 10.1038/s41591-020-0952-y 32546823

[3] Schreyer KE del Portal DA King LJL Blome A DeAngelis M Stauffer K Emergency department management of the covid-19 pandemic. *J Emerg Med.* (2020) 59:946–51. 10.1016/J.JEMERMED.2020.07.022 32948375 PMC7346777

[4] Mitchell R Banks C. Emergency departments and the COVID-19 pandemic: making the most of limited resources. *Emerg Med J.* (2020) 37:258–9. 10.1136/EMERMED-2020-209660 32241814 PMC7211073

[5] Morici N Puoti M Zocchi MT Brambilla C Mangiagalli A Savonitto S. Home-based COVID 19 management: a consensus document from Italian general medical practitioners and hospital consultants in the Lombardy region (Italy). *Eur J Intern Med.* (2021) 84:94–6. 10.1016/j.ejim.2020.11.025 33293151 PMC7709719

[6] Regione L. la Giunta: Deliberazione N° XI / 3876. Determinazioni per la Gestione Integrata Ospedale-Territorio Per L’assistenza dei Pazienti Affetti da COVID 19 o Sospetti. Milano: Gazzetta Ufficiale. (2020).

[7] Ticinesi A Lauretani F Nouvenne A Mori G Chiussi G Maggio M Lung ultrasound and chest x-ray for detecting pneumonia in an acute geriatric ward. *Medicine (Baltimore).* (2016) 95:e4153. 10.1097/MD.0000000000004153 27399134 PMC5058863

[8] Amatya Y Rupp J Russell FM Saunders J Bales B House DR. Diagnostic use of lung ultrasound compared to chest radiograph for suspected pneumonia in a resource-limited setting. *Int J Emerg Med.* (2018) 11:1–5. 10.1186/S12245-018-0170-2/TABLES/2 29527652 PMC5845910

[9] Martínez Redondo J Comas Rodríguez C Pujol Salud J Crespo Pons M García Serrano C Ortega Bravo M Higher accuracy of lung ultrasound over chest X-ray for early diagnosis of COVID-19 pneumonia. *Int J Environ Res Public Health.* (2021) 18:3481. 10.3390/IJERPH18073481 33801638 PMC8037158

[10] Allinovi M Parise A Giacalone M Amerio A Delsante M Odone A Lung ultrasound may support diagnosis and monitoring of COVID-19 pneumonia. *Ultrasound Med Biol.* (2020) 46:2908–17. 10.1016/J.ULTRASMEDBIO.2020.07.018 32807570 PMC7369598

[11] Volpicelli G Lamorte A Villén T. What’s new in lung ultrasound during the COVID-19 pandemic. *Intensive Care Med.* (2020) 46:1445–8. 10.1007/S00134-020-06048-9 32367169 PMC7196717

[12] Yin W Zou T Qin Y Yang J Li Y Zeng X Poor lung ultrasound score in shock patients admitted to the ICU is associated with worse outcome. *BMC Pulm Med.* (2019) 19:1. 10.1186/S12890-018-0755-9/TABLES/6 PMC6318853 30606165

[13] Song G Qiao W Wang X Yu X. Association of lung ultrasound score with mortality and severity of COVID-19: a meta-analysis and trial sequential analysis. *Int J Infect Dis.* (2021) 108:603–9. 10.1016/J.IJID.2021.06.026 34146693 PMC8266421

[14] Circolare Aiop. COVID-19 – Funzione Assistenziale e Incremento Tariffario per le Attività Rese a Pazienti Affetti da COVID-19 – Art.4 D.L 34/2020 – Decreto Interministeriale. (2021). Rome: Gazzetta Ufficiale.

[15] Decreto interministeriale. *Remunerazione di Una Funzione Assistenziale e di un Incremento Tariffario per le Attività rese a Pazienti Affetti da COVID-19.* (2021). Rome: Gazzetta Ufficiale.

[16] De Lorenzo R Conte C Lanzani C Benedetti F Roveri L Mazza MG Residual clinical damage after COVID-19: a retrospective and prospective observational cohort study. *PLoS One.* (2020) 15:e0239570. 10.1371/journal.pone.0239570 33052920 PMC7556454

[17] Breiman L Friedman JH Olshen RA Stone CJ. Classification and regression trees. *Classif Regres Trees.* (2017) 1–358.

[18] De Lorenzo R Magnaghi C Cinel E Vitali G Martinenghi S Mazza MG A nomogram-based model to predict respiratory dysfunction at 6 months in non-critical COVID-19 survivors. *Front Med.* (2022) 9:781410. 10.3389/FMED.2022.781410 35280880 PMC8904385

[19] Quiroz-Juárez MA Torres-Gómez A Hoyo-Ulloa I de León-Montiel RDJ U’Ren AB. Identification of high-risk COVID-19 patients using machine learning. *PLoS One.* (2021) 16:e0257234. 10.1371/journal.pone.0257234 34543294 PMC8452016

[20] IndiniAAscheleCCavannaLClericoMDanieleBFiorentiniG Reorganisation of medical oncology departments during the novel coronavirus disease-19 pandemic: a nationwide Italian survey. *Eur J Cancer.* (2020) 132:17–23. 10.1016/J.EJCA.2020.03.024 32311643

[21] Danese S Ran ZH Repici A Tong J Omodei P Aghemo A Gastroenterology department operational reorganisation at the time of covid-19 outbreak: an Italian and Chinese experience. *Gut*. (2020) 69:981–3. 10.1136/gutjnl-2020-321143 32299837

[22] Jin P Park H Jung S Kim J. Challenges in urology during the COVID-19 pandemic. *Urol Int.* (2021) 105:3–16. 10.1159/000512880 33227808 PMC7801979

[23] de Sutter A Llor C Maier M Mallen C Tatsioni A van Weert H Family medicine in times of ‘COVID-19’: a generalists’ voice. *Eur J Gen Pract.* (2020) 26:58–60. 10.1080/13814788.2020.1757312 32349550 PMC7241505

