# Supplementary material for: A Pilot Study of the Efficacy and Economical Sustainability of Acute Coronavirus Disease 2019 Patient Management in an Outpatient Setting
Source: Front Med (Lausanne). 2022 Apr 27;9:892962. doi: 10.3389/fmed.2022.892962 (PMC9092828; doi:10.3389/fmed.2022.892962)
Supplement: Supplementary file 1 [file Table_1.docx]

Supplementary Table 1. Detailed costs of one-year activity at the Mild-to-Moderate COVID-19 Outpatient Clinic (MMCO).

| *Personnel* | Minutes per visit (min) | Number of visits | Cost per visit (€) | Total cost (€) |
| --- | --- | --- | --- | --- |
| Physician/s | 40 | 1101 | 50.00 | 55,050 |
| Nurse/s | 40 | 1101 | 28.00 | 30828 |
| Clerk | 5 | 1101 | 1.5 | 1651.5 |
| **Total cost of personnel** | | | | **87,529.5** |
|  |  |  |  |  |
| *Consumables* | | | | Total cost (€) |
| Sanitary ware | | | | 21,851 |
| Personal protection equipment (PPE) | | | | 12,464 |
| Stationery | | | | 160 |
| **Total cost of consumables** | | | | **34,475** |
|  | | | |  |
| *Direct structural costs* | | | | Total cost (€) |
| Electrical and water utilities | | | | 5,171 |
| Contracts | | | | 3,177 |
| Maintenance | | | | 4,291 |
| General expenditures | | | | 161 |
| Depreciation | | | | 3,031 |
| **Total cost of direct structural expenditures** | | | | **15,831** |
|  | | | |  |
| *Indirect structural costs* | | | | Total cost (€) |
| **Total cost of indirect structural expenditures** | | | | **14,124.55** |
|  | | | |  |
| **Total cost** | | | | **151,960.05** |
