# Supplementary figures and images for: A Pilot Study of the Efficacy and Economical Sustainability of Acute Coronavirus Disease 2019 Patient Management in an Outpatient Setting
Source: Front Med (Lausanne). 2022 Apr 27;9:892962. doi: 10.3389/fmed.2022.892962 (PMC9092828; doi:10.3389/fmed.2022.892962)

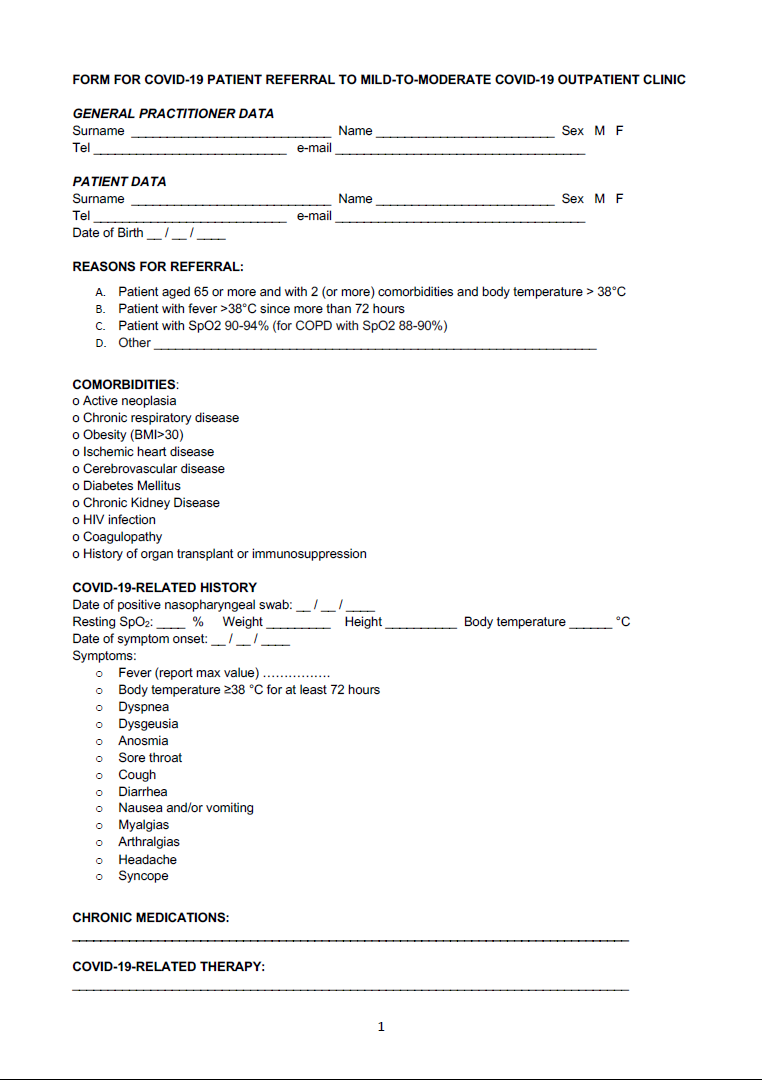

Supplement: Supplementary Figure 1 — Form of patient referral to the Mild-to-Moderate COVID-19 Outpatient Clinic by General Practitioners. SpO2, peripheral oxygen saturation; BMI, body mass index; HIV, Human Immunodeficiency Virus. [file Image_1.PNG]

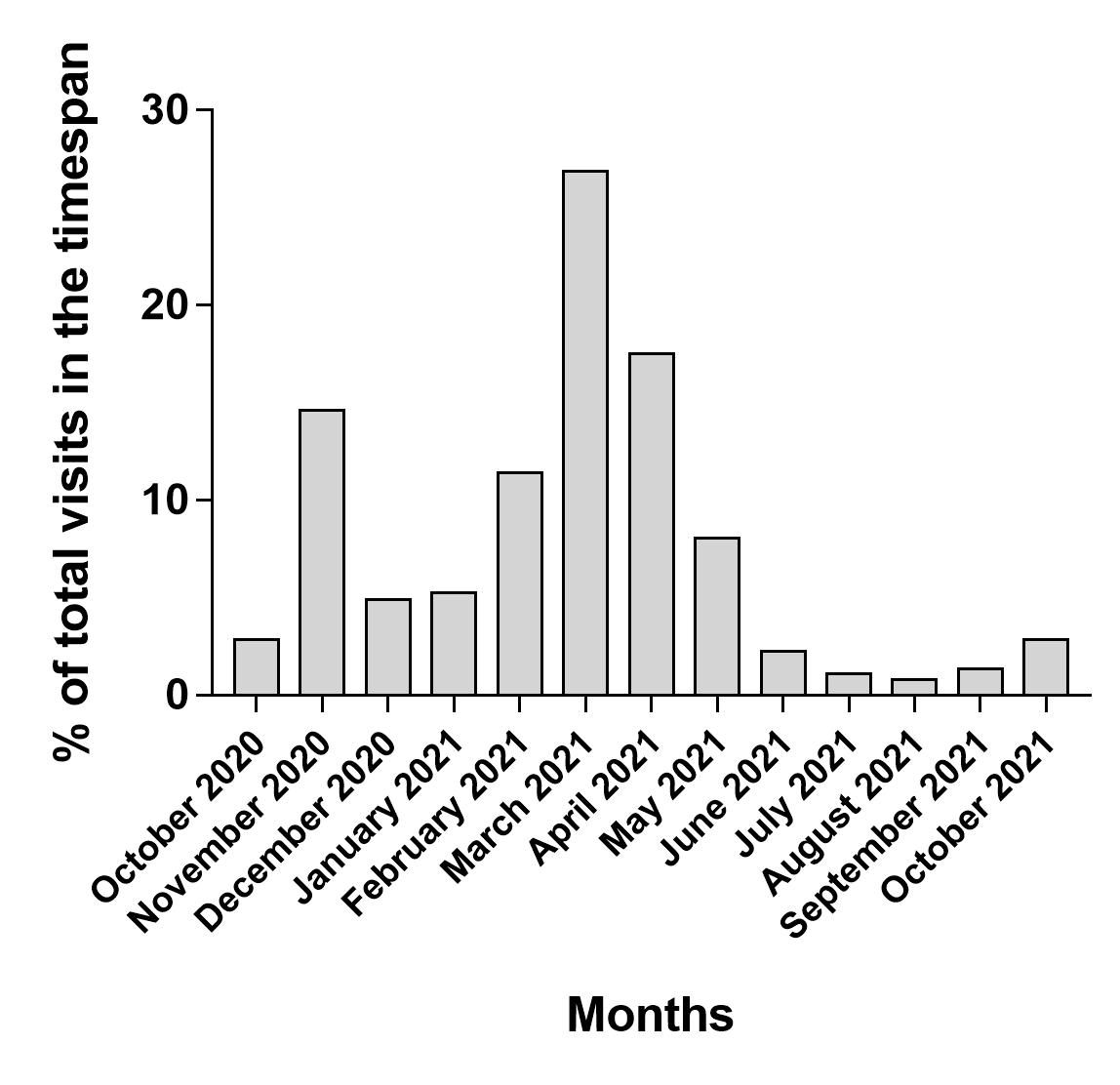

Supplement: Supplementary Figure 2 — Distribution over time of COVID-19 patient visits at Mild-to-Moderate COVID-19 Outpatient Clinics. [file Image_2.JPEG]
